# Committee on Hygiene

**Published:** 1889-11

**Authors:** 


The Committee on Hygiene of the Medical Society of the State of New York, Dr. E. V. Stoddard, of Rochester, Chairman, has issued the following circular note:

MEDICAL SOCIETY OF THE STATE OF NEW YORK.

COMMITTEE ON HYGIENE.

June 1, 1889.

During the past two years, considerable discussion, as to means for increasing the efficiency of administration of local Boards of Health in this State, has resulted in a growing conviction that a change in their organization is necessary to ensure it. The Committee on Hygiene of this Society, at its last annual meeting, was instructed to report on this subject at the next annual meeting, in February.

The suggestion which this Committee would make, is the adoption of the *county* as the *unit of organization*, and not the *city* or *village*, as under the present order, making the county seat the sanitary center, thus reducing the number of local Boards; also making the position of Health Officer the prominent one of the Board, and ensuring capacity for the work, with permanency in office and a sufficient compensation.

The following questions for reply by each County Society, are offered:

1. Are the local Boards of Health of your County as efficient and vigilant as desirable; if not, what are the apparent causes?

2. Would reduction of the number of Boards, by adopting the County as the organizing unit, give better results.

3. Would the change in the position and circumstances of Health Officers, by the removal of political influences, as far as possible, and ensuring permanency in office with adequate compensation, tend to greater efficiency?

Replies to these questions, as much at length as possible, with added suggestions, are specially desired from each County Society by this Committee.

All replies should be sent to the Chairman, E. V. Stoddard, M. D., Rochester, N. Y., as early as possible, and not later than January 10, 1890.

E. V. Stoddard, M. D.,

A. N. Bell, M. D.,

Wm. B. Brown, M. D.,

J. P. Crevelling, M. D.,

Wm. H. Bailey, M. D.,

Wm. C. Bailey, M. D.,

E. F. Brush, M. D.,

*Committee.*

It is also desired that as many individual replies to the above as possible be sent to the Committee at an early date, without regard to the limit of time named. We hope that all who see this will consider it as specially addressed to them, and respond to this important matter with cheerful promptitude. That the efficiency of the local Boards of Health throughout the State needs increasing, goes without question; that the proper medium for the profession to move through in this important matter is the Committee on Hygiene of the State Medical Society, and so to the State Board of Health, and on to the Legislature, goes equally without question. If we ever attain a degree of excellence in the field of preventive medicine, we must, as a profession, strengthen and amplify the powers of our local Boards of Health, and, moreover, yield them cordial and loyal support in the discharge of their functions.

